# Validation of the Mirai model for predicting breast cancer risk in Mexican women

**DOI:** 10.1186/s13244-024-01808-3

**Published:** 2024-10-10

**Authors:** Daly Avendano, Maria Adele Marino, Beatriz A. Bosques-Palomo, Yesika Dávila-Zablah, Pedro Zapata, Pablo J. Avalos-Montes, Cecilio Armengol-García, Carmelo Sofia, Margarita Garza-Montemayor, Katja Pinker, Servando Cardona-Huerta, José Tamez-Peña

**Affiliations:** 1 School of Medicine and Health Sciences, Tecnologico de Monterrey, Monterrey, Nuevo León México; 2 Department of Biomedical Sciences and Morphologic and Functional Imaging, Policlinico Universitario “G. Martino,” University of Messina, Messina, Italy; 3 School of Engineering and Sciences, Tecnologico de Monterrey, Monterrey, Nuevo León México; 4 Department of Breast Imaging, TecSalud, Monterrey, Nuevo León México; 5 Department of Radiology, Memorial Sloan Kettering Cancer Center, New York, NY USA

**Keywords:** Breast neoplasms, Risk assessment, Artificial intelligence, Early detection of cancer, Mammography

## Abstract

**Objectives:**

To validate the performance of Mirai, a mammography-based deep learning model, in predicting breast cancer risk over a 1–5-year period in Mexican women.

**Methods:**

This retrospective single-center study included mammograms in Mexican women who underwent screening mammography between January 2014 and December 2016. For women with consecutive mammograms during the study period, only the initial mammogram was included. Pathology and imaging follow-up served as the reference standard. Model performance in the entire dataset was evaluated, including the concordance index (C-Index) and area under the receiver operating characteristic curve (AUC). Mirai’s performance in terms of AUC was also evaluated between mammography systems (Hologic versus IMS). Clinical utility was evaluated by determining a cutoff point for Mirai’s continuous risk index based on identifying the top 10% of patients in the high-risk category.

**Results:**

Of 3110 patients (median age 52.6 years ± 8.9), throughout the 5-year follow-up period, 3034 patients remained cancer-free, while 76 patients developed breast cancer. Mirai achieved a C-index of 0.63 (95% CI: 0.6–0.7) for the entire dataset. Mirai achieved a higher mean C-index in the Hologic subgroup (0.63 [95% CI: 0.5–0.7]) versus the IMS subgroup (0.55 [95% CI: 0.4–0.7]). With a Mirai index score > 0.029 (10% threshold) to identify high-risk individuals, the study revealed that individuals in the high-risk group had nearly three times the risk of developing breast cancer compared to those in the low-risk group.

**Conclusions:**

Mirai has a moderate performance in predicting future breast cancer among Mexican women.

**Critical relevance statement:**

Prospective efforts should refine and apply the Mirai model, especially to minority populations and women aged between 30 and 40 years who are currently not targeted for routine screening.

**Key Points:**

- The applicability of AI models to non-White, minority populations remains understudied.
- The Mirai model is linked to future cancer events in Mexican women.
- Further research is needed to enhance model performance and establish usage guidelines.

**Graphical Abstract:**

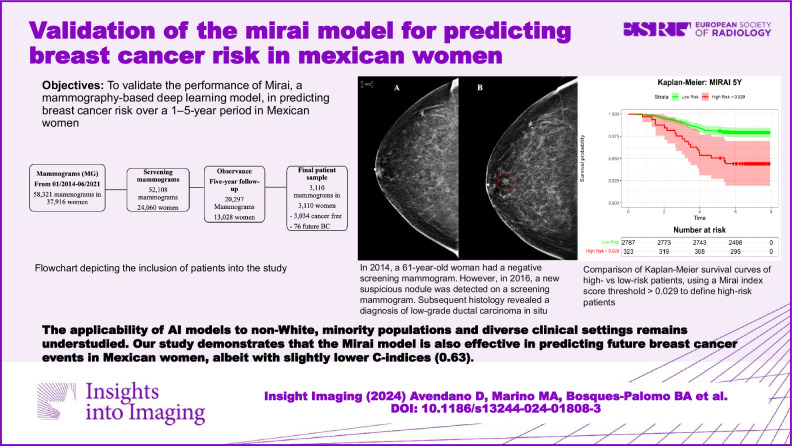

## Introduction

Breast cancer remains the most common cancer among women globally, and early detection is imperative for effective intervention. Opportunistic mammography-based screening programs targeting women aged 40–70 years have been pivotal since the 1980s [1–3], with age as the predominant criterion for screening eligibility [4, 5]. However, in Latin America, where many underprivileged women face barriers to accessing opportunistic screening, the reliance on such screening programs is not as effective as in many high-resource countries and further exacerbates existing social disparities [6–8]. Indeed, breast cancer disparities may be explained by many factors, including access, socioeconomic status, and lifestyle [9]. While substantial advancements in breast cancer detection and intervention have been made over the past three decades, leading to over 40% reduction in mortality rates in certain high-resource countries, low-resource countries continue to face significantly higher mortality rates in comparison to high-resource countries. In low-resource countries, incomplete cancer registries and outdated healthcare technologies, such as analog mammography systems and limited cloud storage, impede the integration of emerging technologies, such as artificial intelligence (AI), that can positively impact and improve healthcare systems in the most affected regions [9].

Prioritizing extensive screening strategies beyond age-based opportunistic screening is essential for early detection and intervention across all age groups in low-resource countries. Personalized, risk-based screening strategies are of particular interest to improve outcomes [10–13]. To date, breast cancer risk assessment has been advanced with various statistical models, allowing patients at high risk of developing breast cancer to be identified and thus undergo earlier and more frequent screening. However, in low-resource countries, specialized clinics to identify and screen high-risk breast cancer patients are lacking, and access to clinical information needed to perform risk assessment using traditional statistical risk models like Tyrer–Cuzick is limited. Moreover, it is important to note that, both in low- and high-resource countries, the use of statistical risk models is not standardized; while physicians, including oncologists and breast surgeons, may use traditional statistical risk models to identify high-risk women, especially those with a family history of breast cancer or other relevant risk factors [14, 15], these models have limitations [16–18] and often overlook genetic variations, lifestyle choices, environmental influences, breast characteristics such as tissue density, and disease heterogeneity [13]. Additionally, most statistical risk models have primarily been tested in White American populations, leading to overestimation or underestimation and hence reduced predictive accuracy in diverse non-White and minority populations [14–18]. Therefore, it is crucial to validate the accuracy and reliability of alternative, accessible tools and models for breast cancer risk assessment in diverse groups [19, 20].

In recent years, AI models like the Mirai model have shown promise for breast cancer risk assessment [21]. However, it is important to note that thorough evaluation across diverse populations is crucial for responsible AI implementation [19, 22–25]. The Mirai model, a mammography-based deep learning model trained on a large dataset from Massachusetts General Hospital, has been validated internationally, outperforming traditional statistical models and previous deep learning risk models across datasets [21, 26]. However, it has not been tested in a primarily Latin American, non-US-born Hispanic population. Moreover, it is worth noting that Mexicans are ethnically recognized as Hispanic/Latin American, mainly of mixed European and Indigenous ancestry with Spanish descent, while Brazilians, also in Latin America and who were included in a previous Mirai study validation, are not considered Hispanic, with demographics comprising 47.7% White and 43.1% of mixed African and European ancestry [27]. Additionally, while Mirai has shown high accuracy in identifying high-risk patients, its evaluation has mainly involved mammograms from Hologic units [21, 26].

Our main objective was to validate Mirai’s performance in predicting breast cancer risk over a 1–5-year period in Mexican women.

## Methods

### Study sample and data collection

This retrospective study received approval from the local institutional ethical committee (protocol number: P000542-MIRAI-MODIFICADO-CEIC-CR002). The local institutional ethical committee also provided a waiver for written informed consent.

A comprehensive retrospective review of the digital mammography database was conducted at TecSalud, a private hospital-based breast cancer center in Mexico. The study identified 58,321 consecutive mammography examinations (both diagnostic and screening examinations) in 37,916 women who voluntarily underwent mammography from January 2014 to June 2021. Subsequently, this was narrowed down to 20,297 screening mammography examinations from January 2014 to December 2016 (to ensure that all included women had at least 5 years of follow-up) in 13,028 women. Women were excluded if they were younger than 40 years, had an incomplete mammography examination (missing at least one of the four conventional views), lacked 5-year follow-up data, or had a mammography examination categorized as Breast Imaging Reporting and Data System (BI-RADS) 5 (high suspicion of malignancy). While patients could have undergone more than one mammography examination during the study period, only the first mammography examination was included in our analysis. After exclusions, the final study sample comprised 3110 screening mammography examinations in 3110 women. We included patients regardless of their history of prior breast surgery, presence of breast implants, atypical lesions, or a personal history of previous cancer. Mammography examinations were performed using either a Lorad Selenia mammography unit (Hologic, New Rochelle, USA) or a Giotto Tomo mammography unit (IMS, Sasso Marconi, Italy). Cancer outcomes (i.e., cancer-positive vs. cancer-negative case) and tumor characteristics were obtained from the institutional tumor registry and from medical records.

### Determining a cancer-positive and cancer-negative case

All cases had a follow-up period of at least 5 years. Cancer-positive cases were defined as cases with a diagnosis of either invasive breast carcinoma or ductal carcinoma in situ within the follow-up period, confirmed through pathology. For cancer-negative cases, screening-based follow-up criteria were used to confirm the absence of cancer within the follow-up period.

### Risk prediction using the Mirai model

The Mirai model has been made freely available as an open-source tool for research purposes. We applied the model to mammographic examinations of the patients in our study to predict each patient’s risk of developing cancer within the follow-up period following their mammographic examination. No image processing was performed. Only image anonymization was carried out by removing patient names while preserving essential information of the image (size, resolution, view, and laterality) [21].

The Mirai model, akin to the Tyrer–Cuzick or Gail model, provides a continuous risk index designed to predict risk rather than diagnostic outcomes. Unlike clinic-based diagnostic tools, Mirai lacks a predefined cutoff value and relies on recommended guidelines for application. A crucial aspect is assessing Mirai’s correlation with true and censored events, often quantified using the concordance index (C-index), a key metric for evaluating predictive accuracy.

### Statistical analysis

For the Mirai model, model performance was evaluated using the C-index. We also determined the area under the receiver operating characteristic curve (AUC) which is typically used in the literature to assess model performance. Additionally, we determined the model’s sensitivity, specificity, positive predictive value (PPV), negative predictive value (NPV), diagnostic accuracy, and relative risk (RR) [28, 29]. For displaying and analyzing receiver operating characteristic curves, the pROC package (version 1.18.2) in R was used.

The calibration of the Mirai model (i.e., how well the model’s predicted outcome frequencies agree with the actual observed outcome frequencies) was evaluated using the observed rate / expected rate (O/E) ratio.

For comparing the performance of the Mirai model between two different mammography imaging systems, the DeLong test was used to test for significant differences between AUCs.

Correlation between breast density, BI-RADS category assessment, and Mirai 5-year risk score was also assessed using Kendall’s Tau statistic (*τ*).

Finally, the clinical usefulness of Mirai continuous risk index’ was assessed by determining the high-risk threshold, following the standard practice of identifying the top 10% of patients in the at-risk category. Subsequently, RR values, confusion matrices, and Kaplan–Meier survival curves were computed for patients in the high-risk group versus others, considering cancer events and time to event development. The significance of differences in Kaplan–Meier survival curves between the high-risk group and others was tested using the log-rank test [30].

All statistical analyses were done in R (version 4.2.3, The R Foundation for Statistical Computing). *p*-values < 0.05 were considered significant.

## Results

### Patient sample characteristics

**Fig. 1:**
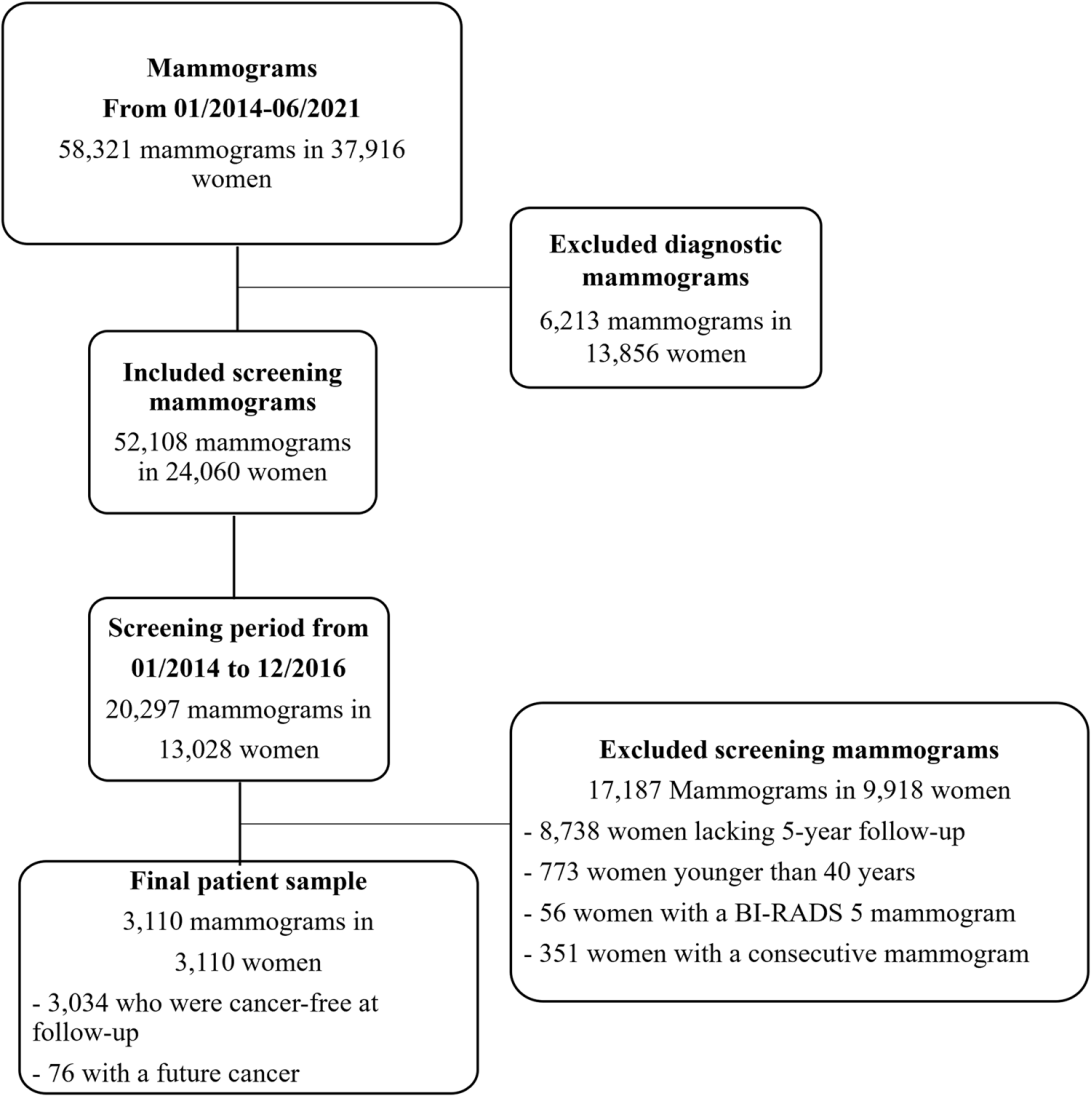
Flowchart depicting the inclusion of patients into the study. BC, breast cancer; BI-RADS, Breast Imaging Reporting and Data System; MG, mammograms; *n*, number of mammography studies

**Fig. 2:**
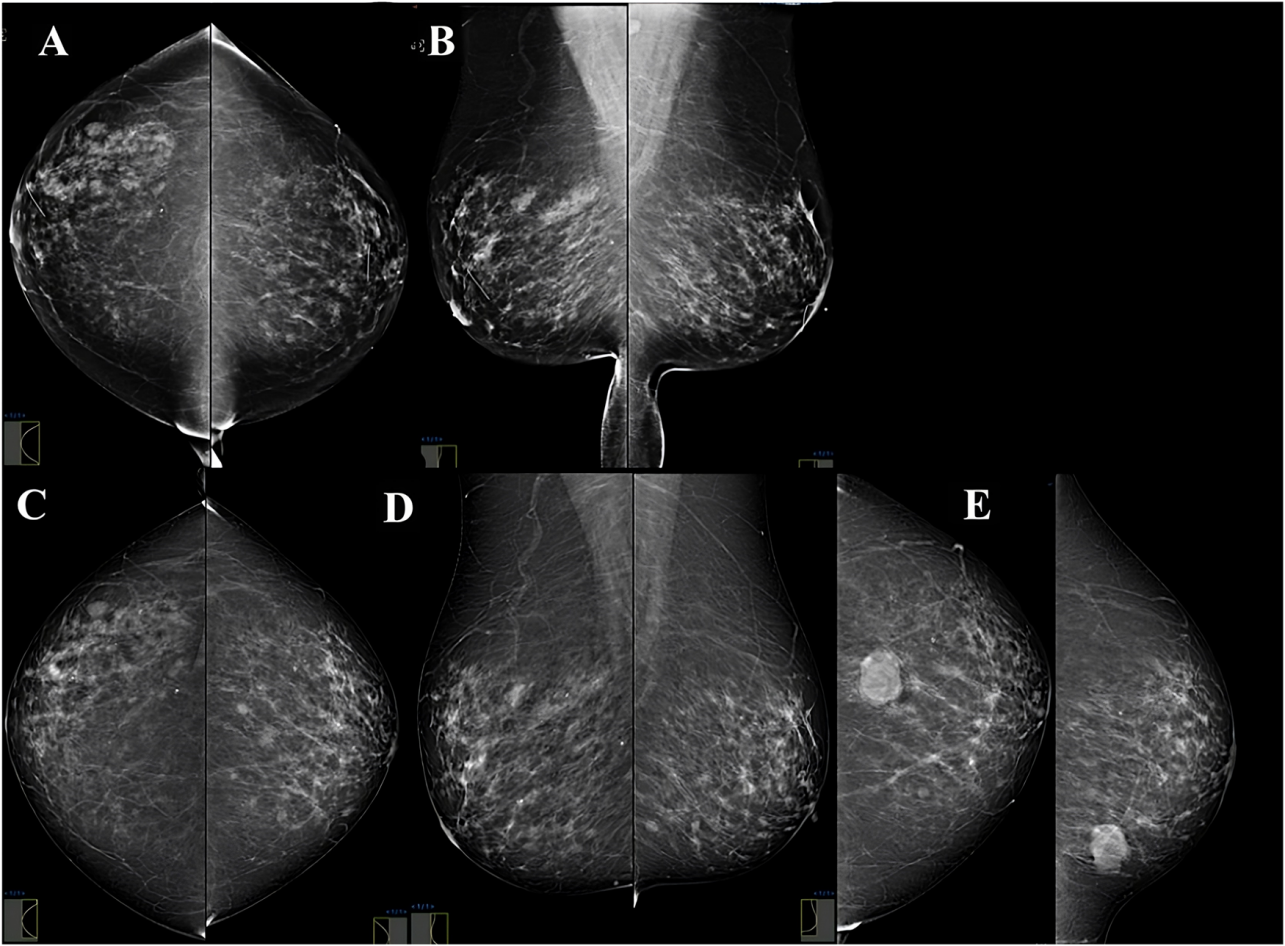
Example of a true-positive case where Mirai correctly predicted a future interval cancer. Bilateral digital mammograms (craniocaudal and mediolateral oblique views) of a 51-year-old woman. In 2014, screening mammography yielded a negative result (BI-RADS 2—benign findings) (**A**, **B**); the Mirai score for this screening mammography examination was 0.084. In 2018, a new suspicious nodule appeared (BI-RADS 3—probably benign findings) (**C**, **D**). Six-month follow-up (**E**) showed significant growth of the previously detected suspicious nodule (BI-RADS 5—highly suggestive of malignancy). Histology confirmed triple-negative breast cancer

From January 2014 to December 2016, a total of 3110 patients (median age 52.6 years ± standard deviation (SD) of 8.9) underwent screening mammography and were included in the analysis. Among these patients, 3034 (98%) (median age 51.8 years ± 8.7) remained cancer-free over the 5-year follow-up period, while 76 (2%) (median age 55.1 years ± 9.7) were diagnosed with breast cancer (Fig. 1). The mean time to a cancer diagnosis was 3 years, ranging from a minimum of 4.5 months and maximum of 6 years. Table 1 gives the characteristics of the entire patient sample, including age, breast density, personal history of breast cancer, family history of breast cancer, and BI-RADS category. Table 2 presents the characteristics of the 76 breast cancers, including histology. Three examples of cancer-positive cases are presented in Fig. 2 and Supplementary Figs. S1, S2 (Electronic Supplementary Material).

**Table 1:** Characteristics of the patient sample at the time of the initial mammographic study

	Total patients analyzed (*n* = 3110)	Cancer-negative cases (*n* = 3034)	Cancer-positive cases (*n* = 76)
Age in years (median ± SD [range])	52.6 ± 8.9 [40–90]	51.8 ± 8.7 [40–90]	55.1 ± 9.7 [40–73]
40–49 years	1428	1402	26
50–59 years	1069	1044	25
60–69 years	487	468	19
70–79 years	114	109	5
> 80 years	12	11	1
Breast density^a^			
No-dense (A, B)	1132 (36%)	1108 (37%)	24 (32%)
Dense (C, D)	1978 (64%)	1926 (63%)	52 (68%)
Personal history of breast cancer			
No	2680 (86.2%)	2615 (86.2%)	65 (85.5%)
Yes	430 (13.8%)	419 (13.8%)	11 (14.5%)
Family history of breast cancer (in a first-degree relative or in ≥ 3 second-degree relatives)			
No	2601 (83.6%)	2539 (83.7%)	62 (81.6%)
Yes	509 (16.4%)	495 (16.3%)	14 (18.4%)
BI-RADS category			
BI-RADS 0	605 (19%)	583 (19%)	22 (29%)
BI-RADS 1, 2	2505 (81%)	2451 (81%)	54 (71%)
Mammography unit			
Hologic	2425	2360	65
IMS	685	674	11

^a^ Breast density was assessed according to the 5th edition of the American College of Radiology Breast Imaging Reporting and Data System (BI-RADS) atlas, whereby breasts are classified into one of four breast density categories: A = almost entirely fatty; B = scattered areas of fibroglandular density; C = heterogeneously dense; and D = extremely dense

**Table 2:** Characteristics of cancer-positive cases within the patient sample (*n* = 76)

Imaging findings and characteristics	Number of cases
Histology	
Invasive ductal carcinoma	46 (60.5%)
Ductal carcinoma in situ	13 (17%)
Invasive lobular carcinoma	6 (8%)
Invasive ductal carcinoma + ductal carcinoma in situ	8 (10.5%)
Other^a^	3 (4%)
Number of years to cancer diagnosis	
0.4^b^–3 years (short term)	34 (44.7%)
< 1 year	4
1–2 years	14
2–3 years	16
> 3–6 years (i.e., long term)	42 (55.3%)
3–4 years	26
4–5 years	9
5–6 years	7

^a^ In the “other” category, there were two mucinous cancers and one tubular cancer

^b^ Refers to a 4-month period

### Performance of the Mirai model

The Mirai model achieved a mean C-index of 0.63 (95% confidence interval (CI): 0.6–0.7) (Table 3), an overall AUC of 0.63 (95% CI: 0.6–0.7) (Supplementary Fig. S3), an overall O/E ratio of 0.99 (95% CI: 0.78–1.25) (*p* = 1.0), and an RR of 2.7 (95% CI: 1.6–4.5). Supplementary Table S1 gives the sensitivity, specificity, PPV, NPV, O/E ratio, and RR of the Mirai model for the prediction of a breast cancer diagnosis in the entire dataset across the entire follow-up period.

**Table 3:** Overview of the performance of the Mirai model across all time points, assessed using the area under the receiving operator characteristic curve (AUC) and the concordance index (C-index)

	Mean C-index (95% CI)	1-year AUC (95% CI)	2-year AUC (95% CI)	3-year AUC (95% CI)	4-year AUC (95% CI)	5-year AUC (95% CI)
Entire patient sample (3110)	0.63 (0.56–0.69)	0.63 (0.56–0.69)	0.63 (0.56–0.69)	0.63 (0.56–0.70)	0.63 (0.56–0.70)	0.63 (0.56–0.70)
Hologic subgroup (2425)	0.63 (0.55–0.70)	0.63 (0.55–0.70)	0.63 (0.55–0.70)	0.63 (0.55–0.70)	0.63 (0.55–0.70)	0.63 (0.55–0.70)
IMS subgroup (685)	0.55 (0.41–0.69)	0.55 (0.42–0.68)	0.54 (0.40–0.69)	0.55 (0.41–0.69)	0.55 (0.41–0.69)	0.55 (0.41–0.69)

When comparing the performance of the Mirai model between the two mammography system subgroups, i.e., the Hologic subgroup and the IMS subgroup, the Mirai model achieved a higher mean C-index for the Hologic subgroup vs. the IMS subgroup (C-index of 0.63 [95% CI: 0.5–0.7] vs. 0.55 [95% CI: 0.4–0.7]) (Table 3). The Mirai model also achieved a higher AUC for the Hologic subgroup (Supplementary Fig. S4) than for the IMS subgroup (Supplementary Fig. S5) (AUC of 0.6 (95% CI: 0.5–0.7) vs. 0.55 (95% CI: 0.4–0.7)), but statistical superiority could not be conclusively asserted due to the limited IMS dataset. Regarding the O/E ratio, the Mirai model achieved an O/E ratio of 0.97 (95% CI: 0.75–1.2; *p* = 0.9) in the Hologic subgroup and an O/E ratio of 0.98 (95% CI: 0.6–1.5; *p* = 1.0) in the IMS subgroup. Supplementary Fig. S6 graphically compares the distribution of the Mirai continuous risk index scores between the two mammography system subgroups.

### Correlation between BI-RADS category and Mirai 5-year risk score

Kendall’s Tau correlation analysis revealed a significant positive correlation between BI-RADS category and Mirai 5-year risk score (*τ* = 0.1; *p* = 2.73 ×10^−^^12^).

### Correlation between breast density and Mirai 5-year risk score

Kendall’s tau correlation analysis revealed a significant positive correlation between breast density and MIRAI 5-year risk score (*τ* = 0.07; *p* = 5.67 × 10^−12^).

### Use of the Mirai model to identify patients at high risk

High-risk threshold values for the Mirai risk index in the entire dataset, in the Hologic subgroup, and in the IMS subgroup were determined by using the top subjects at high risk, with 90% specificity. Using a threshold of 0.029 for the entire dataset, patients in the high-risk group had an RR of 2.68 compared to other patients. Using a threshold of 0.033 in the Hologic subgroup, patients in the high-risk group had an RR of 2.8. On the other hand, using a threshold of 0.017 in the IMS subgroup resulted in a non-significant RR of 1.55 (Supplementary Figs. S7–S9).

Kaplan–Meier disease-free survival plots were generated for high- and low-risk patients. For the entire sample, there were 323 high-risk individuals, 2787 low-risk individuals, and 3034 censored cases with 76 events. Additionally, secondary analyses were performed specifically for patients younger than 55 years and patients 55 years or older. Significant differences were found between high- and low-risk groups (*p* < 0.001), suggesting that the model effectively identified high-risk subjects (Supplementary Figs. S10–S12).

## Discussion

The use of mammography-based deep learning models to predict whether an individual will develop breast cancer in the future has been studied in various populations. Our study aimed to validate the performance of the Mirai model in predicting breast cancer risk over a 1–5-year period in Mexican women. Our findings demonstrate that the Mirai model achieved moderate predictive performance, as indicated by a C-index of 0.63 (95% CI: 0.6–0.7) for the entire dataset. Notably, when comparing performance between Hologic and IMS mammography systems, the Mirai model exhibited a higher mean C-index in the Hologic subgroup (0.63 [95% CI: 0.5–0.7]) compared to the IMS subgroup (0.55 [95% CI: 0.4–0.7]). Furthermore, with a Mirai risk index score > 0.029 (10% threshold) to identify high-risk individuals, the study revealed that individuals in the high-risk group had nearly three times the risk of developing breast cancer compared to those in the low-risk group. The group identified as high-risk at the 5-year exhibited a lower survival rate compared to those at low risk, as evidenced by the Kaplan–Meier curves. These results underscore the potential clinical utility of the Mirai model in stratifying breast cancer risk among Mexican women undergoing screening mammography.

In the multi-institutional validation study of the Mirai model previously published by Yala et al [26], they comprehensively assessed Mirai’s performance across diverse settings in five different countries. Notably, the study primarily used Hologic mammograms, and the Mirai model consistently achieved impressive accuracy (C-indices of 0.69–0.78) across validation sites, excluding recent cancer diagnoses. This highlights Mirai’s potential in utilizing deep learning to enable personalized screening programs with precise breast cancer risk assessments.

In our evaluation of the Mirai model, we found that it achieved moderate predictive performance, as indicated by an overall C-index of 0.63. Additionally, across two distinct mammography systems, namely Hologic and IMS, we observed variations in predictive accuracy. The Mirai model demonstrated superior performance in the Hologic subgroup (C-index: 0.63) compared to the IMS subgroup (C-index: 0.55). Similarly, the model exhibited moderate discriminative ability in the Hologic subgroup (AUC: 0.63), whereas predictive accuracy decreased in the IMS subgroup (AUC: 0.55). However, due to the limited IMS dataset, statistical superiority between the subgroups could not be conclusively determined. Although circumstantial evidence suggests a lack of substantiation for the model’s predictive efficacy in IMS, our study is one of the few investigations exploring the impact of different mammography equipment.

In a recent study, Omoleye et al [31] externally evaluated the Mirai model in a racially diverse population enriched with high-risk individuals, such as those with BRCA mutations or African Americans. Analyzing 6435 screening mammograms from 2096 female patients, the Mirai model demonstrated promising accuracy, with 1- and 5-year AUCs of 0.71 and 0.65, respectively. Our results closely align with those reports, supporting the hypothesis that Latin American women may face a higher-than-average risk and emphasizing the importance of exploring diverse ethnic groups to understand breast cancer risk factors.

When evaluating the ability of the Mirai model to identify high-risk women in our study, those classified above the 90% threshold exhibited significantly shorter survival compared to others (*p* < 0.01), with an RR indicating a threefold higher cancer frequency.

Our study has several limitations, including potential biases in data collection, such as our criteria for selecting patients for inclusion in the study. However, we aimed to accurately represent our clinical environment. Our small sample size, more evident for the IMS system than for the Hologic system, because of a significant lack of data during the 5-year follow-up of the patients, reflects the real-world scenario. Another limitation of our study is that not all mammography system vendors were included, underscoring the need for future research to address differences in performance across systems. Further investigation may be required to validate our findings. Prospective efforts should refine and apply the Mirai model, especially to minority populations and women aged between 30 and 40 years who are currently not targeted for routine screening.

In conclusion, our study demonstrates the moderate effectiveness of the Mirai model in assessing future breast cancer risk among Mexican women. There is a need to optimize its performance in prospective studies and to establish comprehensive usage guidelines.

## Supplementary information

ELECTRONIC SUPPLEMENTARY MATERIAL

## Data Availability

Data generated or analyzed during the study are available from the corresponding author by request.

## Abbreviations

AUC: Area under the curve
BI-RADS: Breast Imaging Reporting and Data System
CI: Confidence interval
NPV: Negative predictive value
O/E: Observed rate/expected rate
PPV: Positive predictive value
RR: Relative risk
SD: Standard deviation

## References

[1] Smith RA, Duffy SW, Gabe R, Tabar L, Yen AM, Chen TH (2004) The randomized trials of breast cancer screening: what have we learned? Radiol Clin North Am 42:793–806 15337416 10.1016/j.rcl.2004.06.014

[2] Duffy SW, Tabar L, Chen HH et al (2002) The impact of organized mammography service screening on breast carcinoma mortality in seven Swedish counties. Cancer 95:458–469 12209737 10.1002/cncr.10765

[3] Tabar L, Vitak B, Chen TH et al (2011) Swedish two-county trial: impact of mammographic screening on breast cancer mortality during 3 decades. Radiology 260:658–663 21712474 10.1148/radiol.11110469

[4] Kumar R, Mardones M, Costa L et al (2023) Beyond October, beyond pink: a year-round revelation for women’s breast health. J Womens Health 32:1143–1146 10.1089/jwh.2023.0663 37787652

[5] Independent UK Panel on Breast Cancer Screening (2012) The benefits and harms of breast cancer screening: an independent review. Lancet 380:1778–1786 10.1016/S0140-6736(12)61611-0 23117178

[6] Pinto JA, Pinillos L, Villarreal-Garza C et al (2019) Barriers in Latin America for the management of locally advanced breast cancer. Ecancermedicalscience 13:897 30792814 10.3332/ecancer.2019.897 PMC6372299

[7] Cazap E (2018) Breast cancer in Latin America: a map of the disease in the region. Am Soc Clin Oncol Educ Book 38:451–456 30231404 10.1200/EDBK_201315

[8] Ginsburg O, Vanderpuye V, Beddoe AM et al (2023) Women, power, and cancer: a Lancet Commission. Lancet 402:2113–2166 37774725 10.1016/S0140-6736(23)01701-4

[9] Fejerman L, Serrano-Gomez SJ, Tamayo LI (2020) Breast cancer risk and mortality in women of Latin American origin. In: Ramirez AG, Trapido EJ (eds) Advancing the science of cancer in Latinos. Springer, Cham, pp 45–55 34460207

[10] Harkness EF, Astley SM, Evans DG (2020) Risk-based breast cancer screening strategies in women. Best Pract Res Clin Obstet Gynaecol 65:3–17 31848103 10.1016/j.bpobgyn.2019.11.005

[11] Pashayan N, Antoniou AC, Ivanus U et al (2020) Personalized early detection and prevention of breast cancer: ENVISION consensus statement. Nat Rev Clin Oncol 17:687–705 32555420 10.1038/s41571-020-0388-9 PMC7567644

[12] Rainey L, van der Waal D, Broeders MJM (2020) Dutch women’s intended participation in a risk-based breast cancer screening and prevention programme: a survey study identifying preferences, facilitators and barriers. BMC Cancer 20:965 33023516 10.1186/s12885-020-07464-2 PMC7539478

[13] Brentnall AR, Cuzick J, Buist DSM, Bowles EJA (2018) Long-term accuracy of breast cancer risk assessment combining classic risk factors and breast density. JAMA Oncol 4:e180174 29621362 10.1001/jamaoncol.2018.0174 PMC6143016

[14] Brentnall AR, Harkness EF, Astley SM et al (2015) Mammographic density adds accuracy to both the Tyrer-Cuzick and Gail breast cancer risk models in a prospective UK screening cohort. Breast Cancer Res 17:147 26627479 10.1186/s13058-015-0653-5 PMC4665886

[15] Brentnall AR, Cuzick J (2020) Risk models for breast cancer and their validation. Stat Sci 35:14–30 32226220 10.1214/19-STS729 PMC7100774

[16] Gail MH (2015) Twenty-five years of breast cancer risk models and their applications. J Natl Cancer Inst 107:djv042 10.1093/jnci/djv042 PMC4651108 25722355

[17] Barke LD, Freivogel ME (2017) Breast cancer risk assessment models and high-risk screening. Radiol Clin North Am 55:457–474 28411673 10.1016/j.rcl.2016.12.013

[18] McCarthy AM, Guan Z, Welch M et al (2020) Performance of breast cancer risk-assessment models in a large mammography cohort. J Natl Cancer Inst 112:489–497 31556450 10.1093/jnci/djz177 PMC7225681

[19] McCarthy AM, Liu Y, Ehsan S et al (2021) Validation of breast cancer risk models by race/ethnicity, family history and molecular subtypes. Cancers (Basel) 14:45 10.3390/cancers14010045 PMC8750569 35008209

[20] Yedjou CG, Sims JN, Miele L et al (2019) Health and racial disparity in breast cancer. Adv Exp Med Biol 1152:31–49 31456178 10.1007/978-3-030-20301-6_3 PMC6941147

[21] Yala A, Mikhael PG, Strand F et al (2021) Toward robust mammography-based models for breast cancer risk. Sci Transl Med 13:eaba4373 10.1126/scitranslmed.aba4373 33504648

[22] van Nijnatten TJA, Payne NR, Hickman SE, Ashrafian H, Gilbert FJ (2023) Overview of trials on artificial intelligence algorithms in breast cancer screening—a roadmap for international evaluation and implementation. Eur J Radiol 167:111087 37690352 10.1016/j.ejrad.2023.111087

[23] Lamb LR, Lehman CD, Gastounioti A, Conant EF, Bahl M (2022) Artificial intelligence (AI) for screening mammography, from the AJR special series on AI applications. AJR Am J Roentgenol 219:369–380 35018795 10.2214/AJR.21.27071

[24] Lokaj B, Pugliese MT, Kinkel K, Lovis C, Schmid J (2024) Barriers and facilitators of artificial intelligence conception and implementation for breast imaging diagnosis in clinical practice: a scoping review. Eur Radiol 34:2096–2109 37658895 10.1007/s00330-023-10181-6 PMC10873444

[25] Chen T, Kharazmi E, Fallah M (2023) Race and ethnicity-adjusted age recommendation for initiating breast cancer screening. JAMA Netw Open 6:e238893 37074714 10.1001/jamanetworkopen.2023.8893 PMC10116360

[26] Yala A, Mikhael PG, Strand F et al (2022) Multi-institutional validation of a mammography-based breast cancer risk model. J Clin Oncol 40:1732–1740 34767469 10.1200/JCO.21.01337 PMC9148689

[27] Centers for Disease Control and Prevention (2022) Health, United States, 2020–2021: Hispanic origin. Available via https://www.cdc.gov/nchs/hus/sources-definitions/hispanic-origin.htm#. Accessed 12 Feb 2024

[28] Longato E, Vettoretti M, Di Camillo B (2020) A practical perspective on the concordance index for the evaluation and selection of prognostic time-to-event models. J Biomed Inform 108:103496 32652236 10.1016/j.jbi.2020.103496

[29] Fan Y, Yin G (2021) Concordance index: surrogacy of progression-free survival for overall survival. Contemp Clin Trials 104:106353 33706004 10.1016/j.cct.2021.106353

[30] Fleming TR, Green SJ, Harrington DP (1982) Performing serial testing of treatment effects. Experientia Suppl 41:469–484 6958531

[31] Omoleye OJ, Woodard AE, Howard FM et al (2023) External evaluation of a mammography-based deep learning model for predicting breast cancer in an ethnically diverse population. Radiol Artif Intell 5:e220299 38074785 10.1148/ryai.220299 PMC10698602

