# Supplementary material for: Validation of the Mirai model for predicting breast cancer risk in Mexican women
Source: Insights Imaging. 2024 Oct 10;15:244. doi: 10.1186/s13244-024-01808-3 (PMC11466924; doi:10.1186/s13244-024-01808-3)

Validation of the Mirai Model for Predicting Breast Cancer Risk in Mexican Women
ELECTRONIC SUPPLEMENTARY MATERIAL

Table S1. Performance metrics of the Mirai model (Mirai index score threshold > 0.029) across the entire dataset.

Metric	Value (95% CI)
**O/E ratio**	0.9 (0.78-1-25)
**RR**	2.7 (1.6-4.5)
**Sensitivity**	24 % (14-34%)
**Specificity**	90 % (88-90%
**Diagnostic Accuracy**	88 % (87-89%
**PPV**	5.5 % (3.3-8.6%)
**NPV**	98 % (97-98%)

Abbreviations: RR, relative risk; HR, Hazard ratio; O/E ratio, observed rate / expected rates; NPV, negative predictive value; PPV, positive predictive value

Figure S1. Example of a cancer with minimal signs on the initial mammographic study. Bilateral Digital mammograms in a 68-year-old woman with a family history of breast cancer (sister) **(A, B)** Negative screening mammogram in 2015, with a Mirai score of 0.015*.* **(C, D)** Bilateral Screening mammogram in 2017 showed in the left breast two hyperdense and irregular masses (red arrows), classified as BI-RADS 5 (highly suggestive of malignancy). Retrospectively, there were two subtle nodules (blue circle) best seen in oblique view **(B)**. Histology yielded a final diagnosis of multifocal invasive ductal carcinoma.

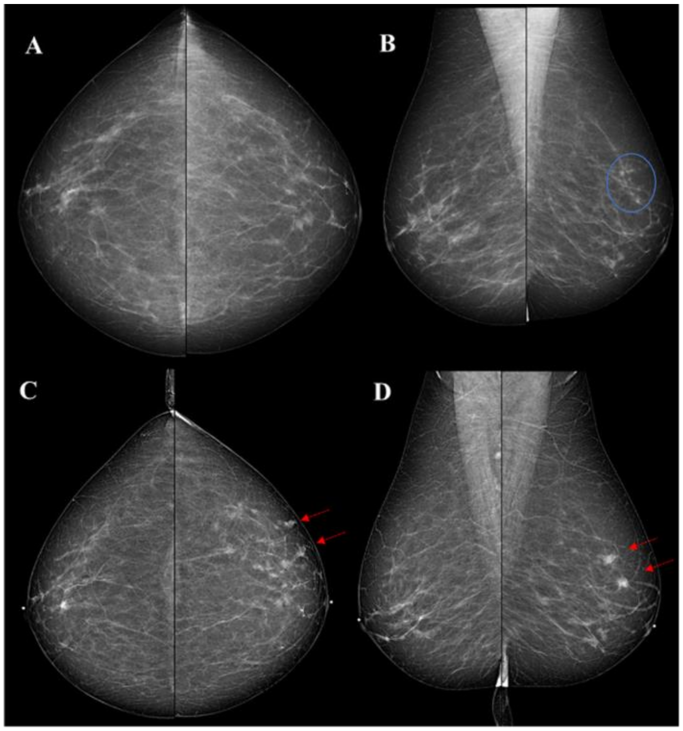

Figure S2. Depiction of a high Mirai risk score in a patient with a history of breast cancer. Digital mammograms (A, B) of a 61-year-old woman with dense breasts and a personal history of left breast cancer (2013). Post-treatment changes were observed in the left breast during the 2014 study, with a Mirai score of 0.030. Subsequent screening mammograms until 2019 (C, D) show no imaging changes and are categorized as BI-RADS 2 (benign findings). No evidence of cancer was found.

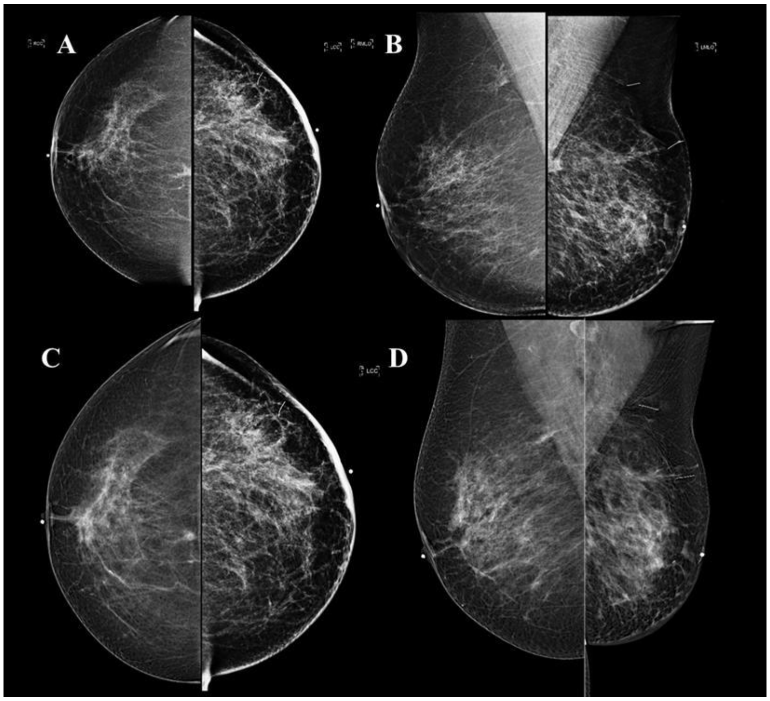

Figure S3. Receiver operating characteristic curve showing the performance of the Mirai model in the entire dataset.

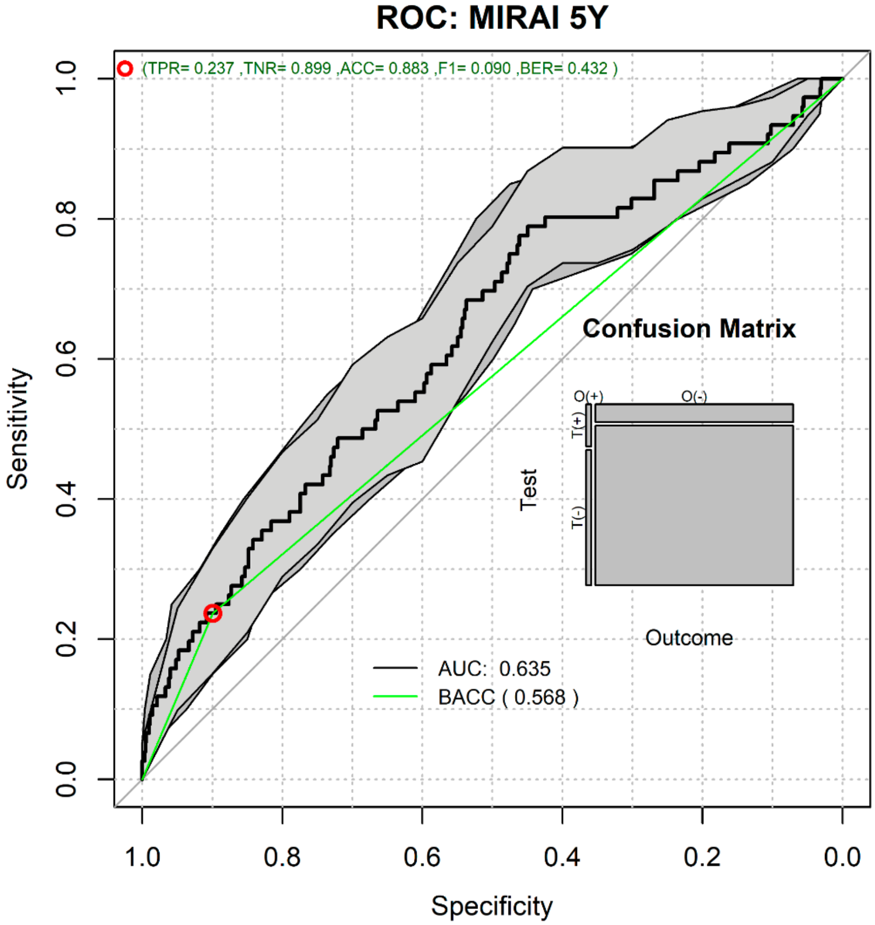

Figure S4. Receiver operating characteristic curve showing the performance of the Mirai model in the Hologic subgroup.

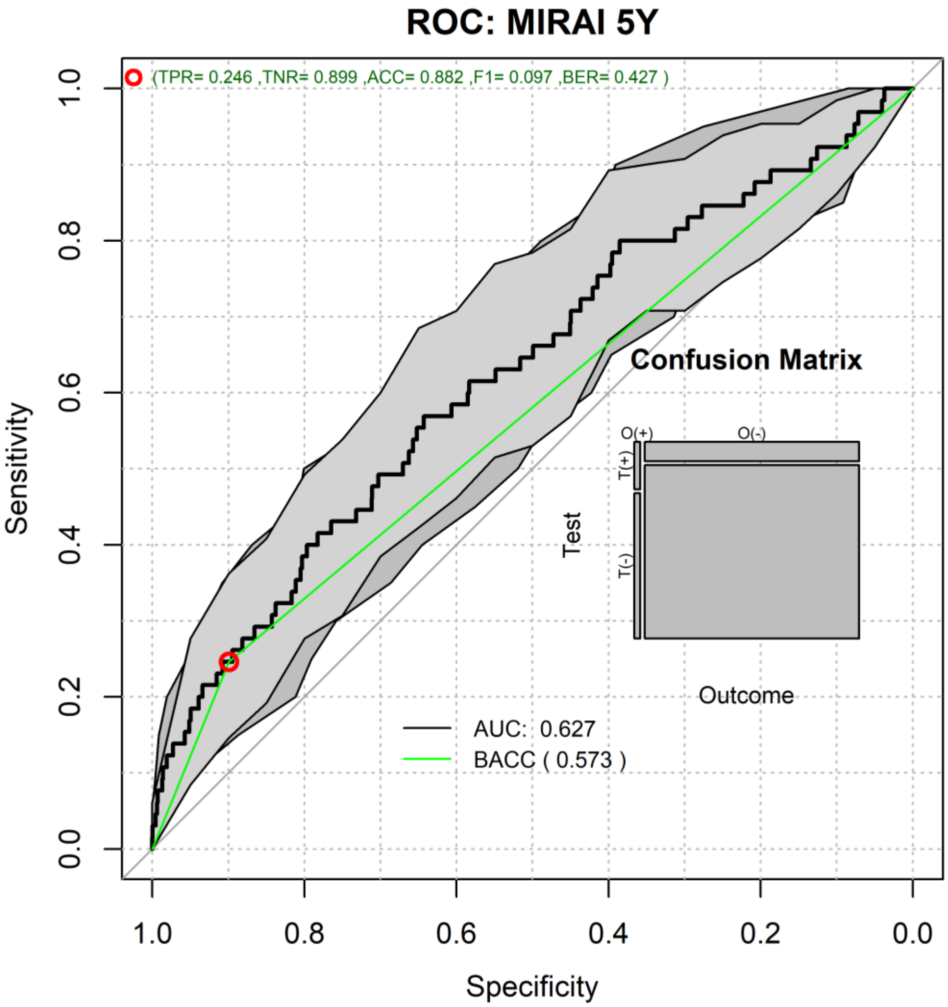

Figure S5. Receiver operating characteristic curve showing the performance of the Mirai model in the IMS subgroup.

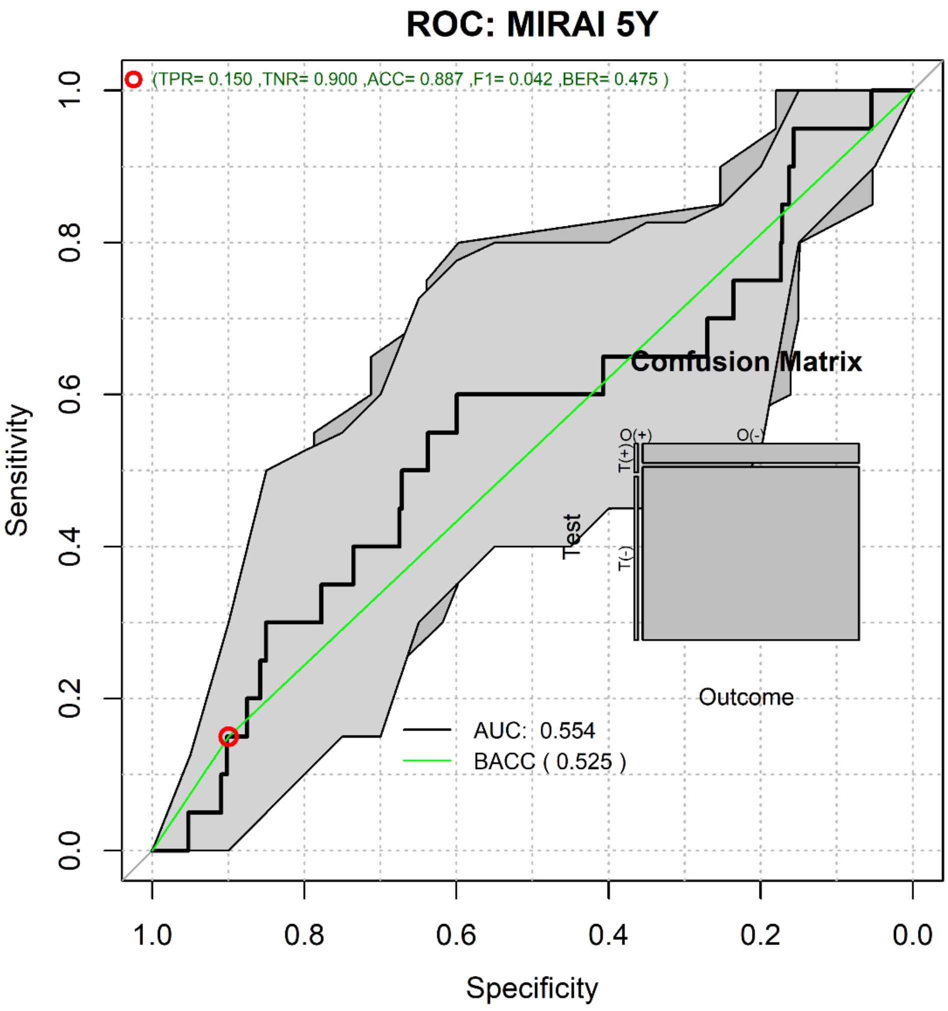

Figure S6: Distribution of the Mirai continuous risk index scores between the Hologic and IMS subgroups.

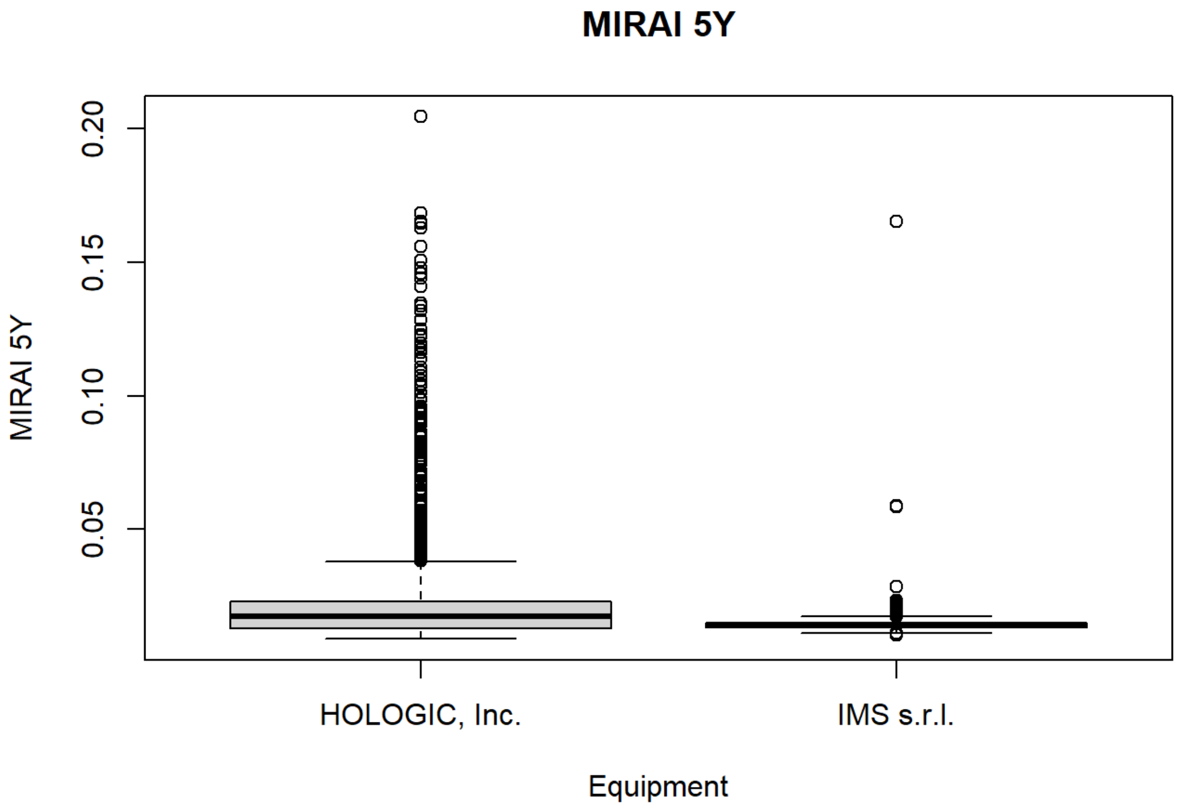

Figure S7. Comparison between the number of positive breast cancer cases across the follow-up period between high-risk patients and low-risk patients in the entire dataset, using a Mirai index score threshold > 0.029 to define high-risk patients.

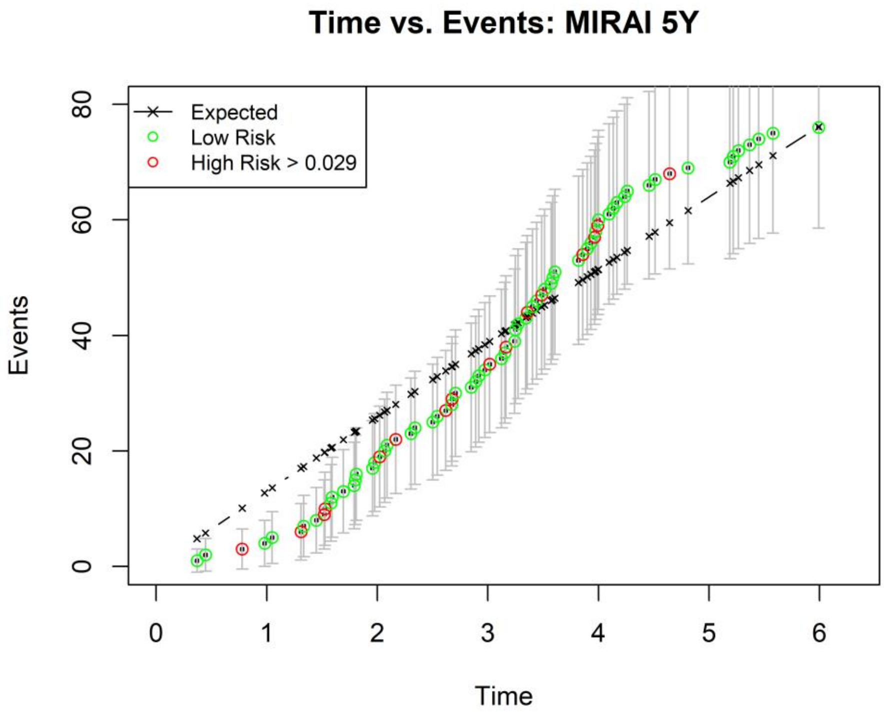

Figure S8. Comparison between the number of positive breast cancer cases across the follow-up period between high-risk patients and low-risk patients in the Hologic subgroup, using a Mirai index score threshold > 0.033 to define high-risk patients.

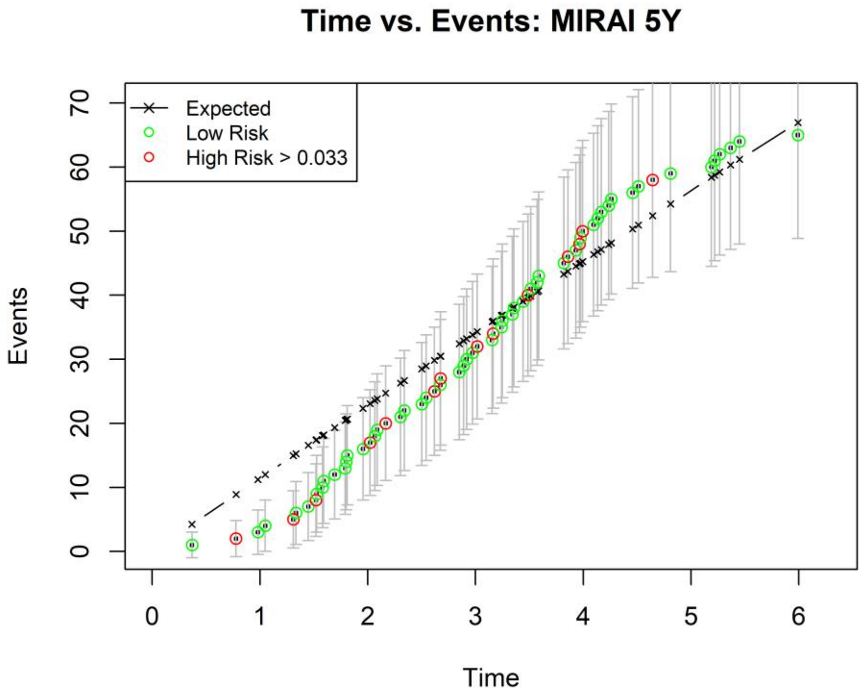

Figure S9. Comparison between the number of positive breast cancer cases across the follow-up period between high-risk patients and low-risk patients in the IMS subgroup, using a Mirai index score threshold > 0.017 to define high-risk patients.

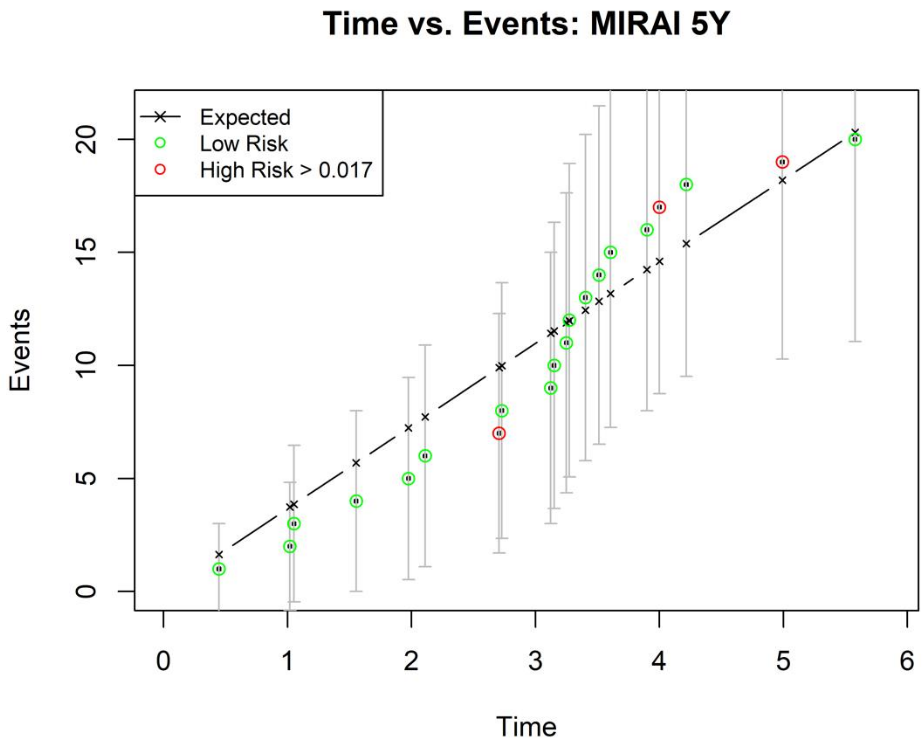

Figure S10. Comparison of Kaplan-Meier survival curves of high- vs low-risk patients, using a Mirai index score threshold > 0.029 to define high-risk patients.

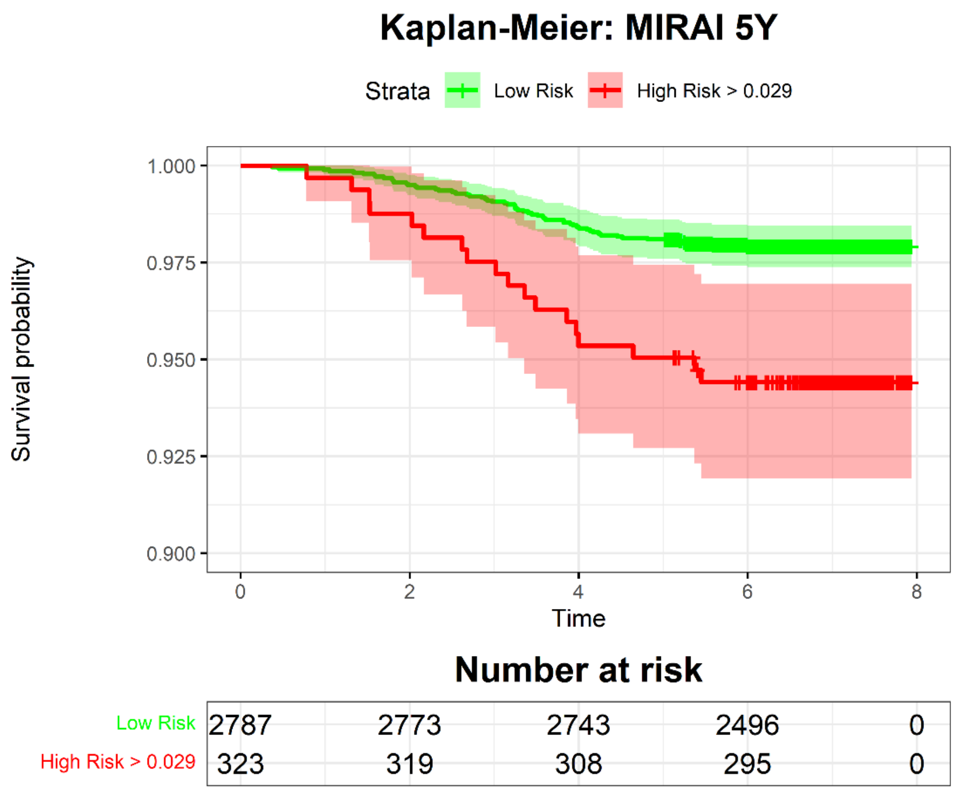

Figure S11. Among patients younger than 55 years of age, comparison of Kaplan-Meier survival curves of high- vs low-risk patients, using a Mirai index score threshold > 0.029 to define high-risk patients.

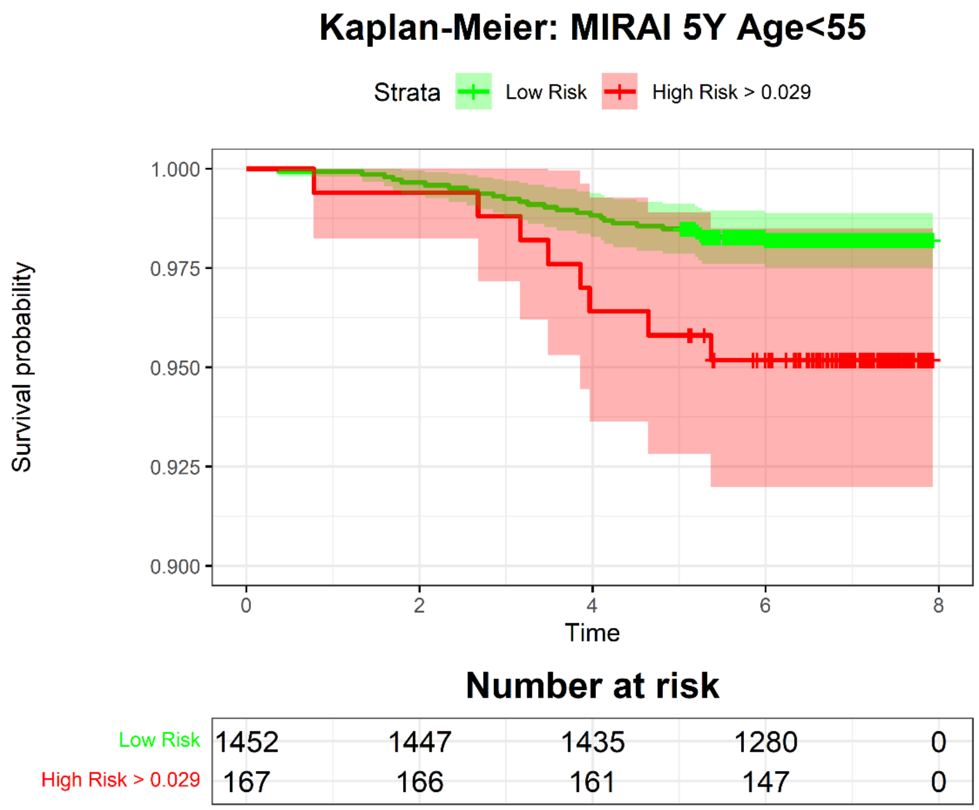

Figure S12. Among patients 55 years of age or older, comparison of Kaplan-Meier survival curves of high- vs low-risk patients, using a Mirai index score threshold > 0.045 to define high-risk patients.

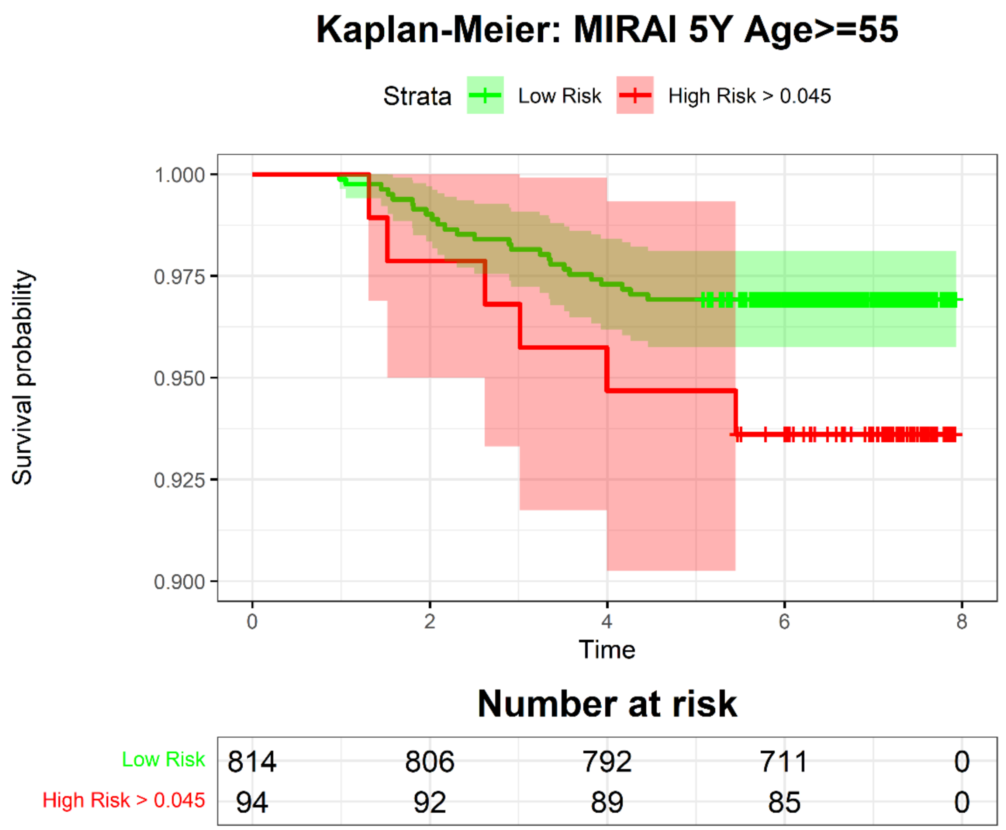

Supplement: Supplementary file 1 — ELECTRONIC SUPPLEMENTARY MATERIAL [file 13244_2024_1808_MOESM1_ESM.pdf]
